# On the Unity or Duality of Syphilis

**Published:** 1873-09

**Authors:** James Nevins Hyde

**Affiliations:** No. 117 South Clark St.; Chicago


ARTICLE VI.—*On the Unity or Duality of Syphilis.* By JAMES NEVINS HYDE, A.M., M.D., Chicago.

Article II of the April number of the *American Journal of the Medical Sciences*, for 1873, is entitled, "On the Present State of the Question of the Unity or Duality of Syphilis, by Freeman J. Bumstead, M.D., of New York." Its author is an eminent syphilographer and a well-known advocate of the tenets of the dualistic school. His opinions are worthy of careful consideration, and the exceeding interest of his subject and his style have placed the article referred to prominently before the profession. After a recital of certain facts which have been brought into notice by some experimenters in Europe, he observes:

"It would be useless to deny that the results of the recent experiments given above, were unexpected by the dualistic school, and cannot be explained by them in accordance with the views which they have held."

I am venturesome enough to believe that these results can be explained, in great part, according to the views held by the dualistic school—*quorum pars sum*—and, in the attempt, shall premise the following statements of some of their views:

1. Every infecting chancre is necessarily followed by syphilis in one who is the subject of it and in another to whom it is transmitted, when no treatment has been pursued.

2. Syphilis has a definite career. It commences with an infecting chancre, but does not recur to an infecting chancre while its energies are unexhausted and not controlled by remedies.

3. Infection having once occurred, all lesions of cutaneous and mucous surfaces become secondary lesions, which present symptoms corresponding to the stage of the disease. These lesions may be transformed chancres, wounds, gonorrhœal inflammation, flea-bites, etc.

4. Secondary lesions are in various degrees contagious. Those are most highly contagious in which occur secretion, elaboration of pus cells, and discharge.

5. An inoculation of syphilitic or other virus, to be successful, must be followed by the characteristic effects of that virus. Other produced effects do not prove that an inoculation has occurred, since they may proceed from causes connected with the constitution of the individual inoculated. Thus a vaccination of a scrofulous subject may produce a scrofulous sore in no way allied to the vesicles of vaccinia.

These propositions stated, I recur to the body of the article referred to. After a short sketch of the origin and progress of the doctrines of the dualists, Dr. Bumstead formulates them in ten paragraphs, which express fully, with one exception to be again considered, the prevailing views of the school. He then describes the "mixed chancre," and proceeds to the subject of Prof. Boeck's visit to England and America. He says:

"In England, Prof. Boeck succeeded in some seven cases, and in my wards at Blackwell's Island, after a number of failures, he met with success in one instance," in inoculating the secretions of true chancres and syphilitic condylomata, with a view to produce syphilization. In other words, Prof. Boeck *occasionally* succeeded in producing secondary lesions upon the skin of syphilitic patients by irritations with a lancet.

"His method was to inoculate every day, and in some instances twice a day, and he stated that success would be finally attained, though possibly not until after the lapse of several weeks."

This is just what the history of consecutive symptoms would teach us to expect. A period of some weeks generally elapses before the system is capable of expressing its infected condition by the exhibition of secondary lesions. This is that "period of incubation" which Dr. Bumstead himself teaches when he says, in his work on Venereal Diseases,* (p. 441), "the earliest symptoms of general syphilis . . . have been preceded by a chancre probably between three and certainly within six months." Nor would the coexistence of a primary sore and the results of these so-called inoculations, negative this statement of the essential nature of the latter, since it is well known that the two conditions are frequently observed to be contemporaneous.

An acceptance of the 5th of the propositions stated at the outset of these remarks, involves a complete disbelief in the production of any real inoculation in the cases operated upon by Prof. Boeck, and a different explanation of the modus operandi of his cures by syphilization. The skin is tattoed† with a lancet—no matter what manner of virus is used—until irritated to such an extent as to exhibit the lesions of secondary syphilis. The first explosion of the constitutional disease is thus made to anticipate somewhat the date of its ordinary appearance, and the effect is to dèlay, or prevent, a further explosion. It is in a similar way that the period of latency of inoculated variola is shortened and its subsequent career modified.

Dr. Bumstead continues by referring to Mr. Henry Lee, of London, who, in 1856, showed that—

"A chancre would become readily auto-inoculable if it was irritated in such a manner as to render its secretion decidedly purulent. This fact was confirmed by Bidenkop, Kobner, and Pick, in Germany, who not only applied powdered savine to the surface of chancres in order to irritate them, as Lee had done, but also accomplished the same purpose by passing a seton of horse-hair through the base of the sore. Similar treatment of syphilitic condylomata had the same effect, and rendered their secretion inoculable, though with greater difficulty."

These conditions are readily explained. But in order to do so satisfactorily, it is necessary to agree, 1st, that contagion is, for all

* The Pathology and Treatment of Venereal Diseases, including the results of recent investigations upon the subject; by Freeman J. Bumstead, M.D. Philadelphia, 1866.

† I use the word advisedly—no other occurs to me equally descriptive of Boeck's procedure. An individual in perfect health could not fail of being profoundly affected by the many hundreds of lancet wounds inflicted in accordance with the Professor's tireless routine.

practical purposes, immediate,* and, 2d, that a chancre is an expression and a result of that contagion; and is as much a con stitutional symptom as a syphilitic node.† Such being the case, what is the result of artificial irritation of a chancre by powdered savine, or by a horse-hair seton? I affirm that it is changed, transformed, converted to a syphilitic accident of another grade. It ceases to be a chancre and becomes a syphilide. Mr. Henry Lee elsewhere confesses that he cannot, at all periods of its existence, produce this change in the secretion of the sore.‡ Why? Simply because it is necessary that a certain evolution of the disease should occur before such a transformation can be effected. Observe that syphilitic condylomata exhibited corresponding changes—a fact which clearly indicates the affinities of the two classes of lesions. Now I have endeavored to lay down in the 4th proposition, that the secretion of a pus cell in a secondary lesion is an element of contagion. If all this be granted, can it be said that the auto-inoculation of these secretions produces a true infection, or that the resulting sores are chancres? Is it not rather true that the irritation of the skin of a syphilitic patient, in whom these secondary changes have been induced, provokes the development of additional lesions of the same general character, and from the same constitutional source? It may be admitted, however, that there is, in these instances, an anticipation of the date of the natural explosion of the disease.

Dr. Bumstead concludes from the foregoing experiments that—

1. "Neither the chancre nor any of its secondary manifestations can readily be inoculated with success upon the persons bearing them, nor upon any other persons under the influence of syphilitic infections.

2. "Successful auto-inoculation, however, of these lesions may be attained by irritating them, by the methods just mentioned, so that their secretion is rendered decidedly purulent.

3. "The effect of the successful inoculations is apparently the same as that produced by the inoculation of chancroidal matter. In the course of about forty-eight hours, or without incubation, a

* Cazenave, Traite des Syphilides, p. 142, et seq. Paris, 1843.

† Lancereaux, Sur la Syphilis, p. 63. Paris, 1866.

‡ Lancet, April, 1866.

pustule appears which covers an ulcer with abrupt edges, a soft base, and a tendency to spread; and which will reproduce itself on artificial inoculation through a number of generations."

The first two of these conclusions do not require comment in view of what has preceded. Of the third, I remark, that according to the latest opinions of dermatologists, a pustule is nothing more nor less than a local abscess on a small scale. A pustule, in a subject of syphilis, is a small local abscess, modified by the constitutional dyscrasia. Do we find such modified abscesses in the skin of patients suffering from syphilitic disease? Let Dr. Bumstead himself answer (op. cit. p. 528). "In the superficial variety" (of pustular syphilis—ecthyma, etc.,) "the scab first formed exposes (on removal) a superficial ulceration. In the deep variety, its (the ulcer's) edges are abrupt, and covered with a grayish secretion." And again (p. 527) in describing the pustules of syphilitic impetigo, he says: "Their base is sunken within a prominent border of the same aspect." Squire* describes these same ulcers as being "moderately deep, having . . . clean-cut edges and a grayish floor."

If any one is disposed to doubt whether such secondary lesions could be contemporaneous with some periods of chancrous erosion, I reply that such a doubt cannot fail to be removed by a consideration of the clinical truth of other facts. The division of syphilis into secondary, tertiary and even quaternary periods is entirely artificial and arbitrary, and, as Mauriac† has pointed out, is no more applicable to syphilis, than to any other constitutional disease. This author gives full details of one case, in which, at the very inception of the disease, so-called tertiary complications involved the bones, periosteum, splanchnic viscera and muscles—another of pericranial periostitis at the very outset of syphilis—another of frontal node, occurring only twenty days after chancre, which was *succeeded* by roseala and secondary cutaneous and mucous accidents—and yet another of periostitis, succeeding doubtful primary disease, and *followed* by papulo-squamous syphilide and laryngopathy.

The words "secondary" and "tertiary" are, however, so gener-

* Manual of Diseases of the Skin, by Balmauno Squire, M.D., F.L.S., p. 147. London, 1868.

† Gazette des Hopitaux, 1872. Charles Mauriac, Physician to the Hopital du Midi, Paris.

ally used and understood, that for convenience merely, they are employed in the course of these remarks in the sense according to which they are commonly accepted.

To return to the remarks of Dr. Bumstead, he continues: ". . . three cases have been reported by Bidenkop as exceptions to this course. In one, an indurated ulcer is said to have been developed after the lapse of two to four weeks; in the other two, papular elevations without induration and becoming only superficially ulcerated, followed after a lapse of four and three weeks respectively; at the same time that pustules appeared from later inoculations, made a few days before, with the same matter and upon the same patients."

These, surely, are the exceptions that prove the rule. No comment is necessary to lead the reader to distinguish clearly, in these results, the influence of a syphilitic infection and its production of secondary lesions.

Now arises the question: What would ensue from an infection of healthy individuals with virus obtained from these sores induced upon the skin of syphilitic subjects—call them secondary lesions, chancres, chancroids, or what you will? If opportunity for such an inoculation were to occur, would you not possess an "experimentum crucis?" To this question the consistent dualist replies: Constitutional syphilis, preceded by infecting chancre, would undoubtedly ensue. Dr. Bumstead, in response, quotes the following cases reported by Bidenkop of Christiania:

"Olive Martinsdalter, seventeen years old, entered the hospital, Oct. 9, 1862, with gonorrhœa of the vagina and urethra. She had recently come from the country, and had never before suffered from any venereal disease. Her health was otherwise perfect. She was treated with alum tampons in the vagina, and a solution of nitrate of silver in the urethra.

"On the 25th of November she inoculated herself on the epigastric region, by means of a needle. The matter was taken from the artificial ulcers of a patient, who was being treated by syphilization, and these ulcers had been produced, many generations back, by the author's inoculation of matter from an infective chancre. She concealed for a week what she had done; but, as the inoculation had succeeded only too well, she was compelled to show it. On examination there was found a round ulcer of the size of a pea, with sharp edges, sunken floor, and surrounded by a reddish swelling. The secretion of the ulcer was copious, and there was no trace of induration.

"She stated that a pustule had appeared a few days after the inoculation, and had terminated in the ulcer. The latter was treated with a water dressing. It soon increased decidedly in size, and the inflammatory swelling became greater, but without becoming perceptibly indurated. By the end of three weeks it had attained a diameter of rather more than four lines; it was tolerably deep, with callous edges, and its secretion had become scanty and thinner. It was now lightly touched with stick nitrate of silver.

"December 28.—A swollen gland of the size of a walnut, somewhat painful on pressure, appeared in the left axilla, but disappeared again in three weeks.

"January 27, 1863.—The ulcer had decreased to the size of a pin. There was not the slightest infection of the system. In consequence of spontaneous inoculation, a small ulcer now formed by the side of the old one, but soon healed.

"March 5.—The patient was dismissed. The ulcers had healed and left behind them, bluish, somewhat elevated scars, which were not indurated. There was no swelling of the glands, and no symptoms of syphilis.

"The patient was subsequently examined nearly every week by the author, who could not discover the slightest trace of constitutional disease.

"In the summer of 1864, however, she contracted a chancre, which was followed in a few months by roseola and other constitutional symptoms."

The second case, reported by Bidenkop, is thus detailed:

"The patient, a girl free from syphilitic antecedents, had entered the hospital for eczema. She inoculated herself with matter taken, as in the first instance, from patients undergoing treatment by syphilization, who had been successfully inoculated, several generations back, with the secretion of their own chancres. As a result of these inoculations, pustules and ulcers appeared, without incubation, and several of them finally left hardish scars. Some symptoms of a suspicious character afterwards appeared on other parts of the body, but were not regarded as syphilitic by the attending physicians."

Three other cases are alluded to, which were communicated by Dr. Gjor of Christiania.

Dr. Bumstead admits that these five cases are "imperfectly reported and open to criticism." As the details are not fully given, I remark of the first, hypothetically:

1. The patient may have been admitted with a syphilitic gonorrhœa, from which she became infected, produced an ulcerating syphilide by her so-called inoculation of Nov. 25, and had further accidents develop in 1864—the character of the "chancre" then contracted having been misunderstood. By "syphilitic gonorrhœa," I mean a purulent discharge contracted from an individual infected with constitutional syphilis. Such a discharge (see Proposition 3,) partakes of the nature of secondary lesions in a syphilitic subject, and is capable of transmitting the disease. To this source of infection agree Dr. Bumstead, (op. cit. p. 72), Tonner,* Erichsen, Fournier and others. Forty-seven days, at least, elapsed between the date of admission of this patient and the appearance of the epigastric ulcer; and syphilogrophers agree that but twenty days† may intervene between the date of infection and that of the outbreak of general syphilis.

2. The inoculated region may have exhibited an infecting chancre, and constitutional disease manifested itself as a result in 1864, the character of the sore which appeared in the summer of that year having been misinterpreted. Cazenave is an authority for the statement that the first explosion of the disease after contagion may be delayed for two years,‡ but in this case it is only necessary to suppose a delay of eighteen months. As to the characteristics of the sore produced by the needle, it is noticeable that for one week the patient concealed it from observation. During that period it may have exhibited a pathognomonic induration. This peculiarity of the initial lesion of syphilis often appears on the third day after infection,§ and, to use the phrase of Dr. Bum-

* Practice of Medicine, by Thomas Hawkes Tonner, M.D., F.L.S., p. 294. Philadelphia, 1872. Also, Erichsen's Science and Art of Surgery, p. 524. Philadelphia, 1869.

† Lancereaux, op. cit., p. 64, and many others.

‡ Op. cit.

§ "On Induration in Syphilis," by Victor de Merie, F.R.C.S., Medical Press and Circular, Feb. 16, 1870. Mr. Merie shows in this paper, 1st, that our stead (op. cit., p. 412), is often "short lived." This would be exceedingly apt to occur in an epigastric chancre, and the rarity of lesions of this character, in such a locality, would render an accurate diagnosis difficult. Of 470 infecting chancres observed at the Hopital du Midi,* one only occurred on the leg and one upon the finger—none on the skin of the body—the field of study therefore in such cases must be limited.

3. On the very showing of Lee, Kobner, and Pick, this epigastric ulcer, situated in a locality extremely favorable for irritation by the clothing, may have been an infecting chancre, whose secretion became copious in consequence of such irritation. The "inflammatory swelling" which subsequently occurred, would point to such a cause. The non-suppurating axillary bubo and the formation of a smaller ulcer in the neighborhood (pustular and encroaching syphilide†) would equally lead to such a conclusion—the roseola of 1864, being, on this hypothesis also, presumed to be a resultant.

Of the second case I remark that the "symptoms of a suspicious character," the "hardish scars," etc., throw so much doubt upon the results of the "inoculation" that any conclusions to be derived from it would prove in a great degree untrustworthy.

Dr. Bumstead continues:

"In 1865, Prof. Pick, taking the matter from pemphigus, acne, scabies and lupus, occurring in persons who had never had syphilis, inoculated it, first, upon the individuals from whom it was taken, without success; but this same matter, inoculated upon syphilitic subjects, gave rise in three instances to pustules not preceded by incubation, and the secretion of which was further inoculated, through many generations. Similar experiments by Reder and Kraus proved successful in one instance."

knowledge as regards the nature of the induration is, as yet, imperfect; 2d, that very striking distinctions exist as to the shape and feel of the induration *according to locality;* 3d, That mistakes are exceedingly likely to occur in distinguishing syphilitic induration from simple œdema and ordinary infiltration. He also demonstrates the *complete absence* of induration in the *irritatory symptoms* of syphilis. Cases are quoted to establish this fact, and exceptional instances are noted where no evident irritatory symptom could be made out.

* Cited by A. Martin, These de Paris, p. 62.

† American Journal of Syphilis and Dermatology, Jan., 1873, p. 58.

Could any stronger proof of the position which I have taken in regard to the secondary character of these induced lesions, be advanced? The very innocuousness of this matter from acne and pemphigus, when introduced into healthy skins, demonstrates that its introduction into the skin of a syphilitic subject, was succeeded by an effect attributable *only* to the syphilitic disease, and proves that the wound of the lancet excited the local disorder. This provocation of secondary lesions is, as the experimenters show, a difficult and rare feat. In four instances only are we informed of its successful accomplishment.

Now if these lesions be not secondary syphilides, and not results of inoculations of a virus, what are they? Will any one contend that they are *non-inoculated* and non-syphilitic chancroids?

Dr. B. continues by referring to the experiments of Mr. Morgan, of Dublin, who has, within a few years, produced "pustules capable of further inoculation through a number of generations, by inoculating syphilitic women with their vaginal secretion."

This also is a result entirely in accordance with facts stated by many authors, to which allusion has been already made. The secretion of a pus cell, either in the vagina or nares of a syphilitic subject, partakes of the character of a contagious secondary lesion, and will inoculate others with the disease, as well as set up an irritation capable of evoking other syphilides in the same subject.

Dr. Bumstead concludes by stating the effect produced on various authorities by the results of the experiments given above. Sigmund and Zeissl remain fixed in the belief of the dualistic school. Reder has "from a scientific standpoint" abandoned its tenets. Auspitz, Hebra, and Michaelis, are in favor of unity. Prof. Pick, of Prague, is non-committal; while Geigel and the great Ricord in France, are enthusiastic advocates of the doctrines of dualism.

Dr. Bumstead's seventh proposition—of the ten in which those doctrines are formulated—is, in part, open to criticism. It reads as follows:

"7. The nearest ganglion in anatomical connection with a chancre almost invariably, and the intervening lymphatics not unfrequently, become indurated at the same time as the chancre itself. If suppuration takes place either in the ganglia or lymphatics, it is due to accidental causes, giving rise to common inflammation ; and the pus is simple pus, destitute of specific quality."

If by this it is meant that the discharge from such a bubo will not inoculate the lips of the opening through which it escapes, and thus give rise to a specific ulcer, as is the case with a bubo originating from a non-infecting sore (chancroid), there can be no question of its accuracy. But if it is meant to teach that there can be any "common inflammation" or "simple pus" in one who is the subject of syphilis, *so far as regards possibility of infecting another*, there is room for dissent. The constitutional dyscrasia in such cases is, without doubt, engrafted upon every morbid process and every morbid product of the system.* It is entirely true, as Dr. Bumstead remarks in conclusion, that "a new field for investigation and experiment has been opened, which no one has as yet fully explored, and no one can pretend to understand." And I am convinced that this investigation can be most satisfactorily conducted, with a complete establishment of the fourth of the propositions stated at the outset of these remarks, viz.: that where there is elaboration of pus cells in a syphilitic subject, there is a possibility of transmitting the disease.

It is not without great diffidence, and a sense of his own unfitness for such a task, that the writer of these lines has attempted to establish the views of an accomplished author, in the face of the facts collected by himself. For the sake of convenience and distinction of statement merely, has the first person been largely employed. It was from the lips of the best syphilographer of America that he learned, some twelve years ago, his first lessons in dualism. And he cannot refrain from comment upon an article which appeared in the *Chicago Medical Examiner* for October, 1871, entitled "The Unity or Duality of the Syphilitic Virus."

After a consideration of several of the results obtained by the German and French experimenters, the author of the article in the *Examiner* concludes with a pointed reference to Ricord, Bassereau and Bumstead. He says:

"The fluctuations of this remarkable controversy are adapted to arouse indignation in a thinking mind. What is the use of science in the hands of men who contrive, not how to bring out

* Aitken's Science and Practice of Medicine ; Vol. 1, p. 694. Philadelphia, 1868.

solid and permanent truth, but only some startling conclusions upon which to base a reputation? Who are these pseudo-great men to whom we have given the highest honors of science for forty years, for telling us, first, that the syphilitic virus is single, then that it is undoubtedly double, then again that it is single, and, finally, that they don't know which it is! Would any block-head have done worse by simple guess-work? There is only one remedy for this foolery, and that is, to leave off honoring men for half-made discoveries, and to scourge with the lash of criticism and contempt, all efforts to place crude and ill-sustained opinions in the rank of ascertained truths."

Without attempting to criticise the rhetoric or spirit of this contemptuous "lash," it may be profitable to remind him that "solid and permanent truth" is most often attained by a process of "half-made discoveries." Rarely is it allotted to one man to subtract all the golden ore from its hidden veins. Piece by piece, with much alloy, through arduous labor and by the aid of fellow-workmen, is it brought to the crucible. Columbus never saw the main-land of this continent, but it is inscribed upon his tomb-stone* that he "gave a new world to Castile." Bichat, Hunter, Paget, and Virchow, have been justly honored, though they gave many half-truths to the world, and often much less than that. And were the doctrines of dualism to be to-morrow irrefutably disproved, they who have toiled and studied and written to set them forth, would be chiefly honored for their contribution to the final result.

No. 117 South Clark St.

---

An Unhealthy Business.—No business is so fatal to life as that of selling liquor, because those who sell usually drink. A recent report in England on the influence of occupation on health, proves that even those who work in mines in the bowels of the earth live longer than liquor sellers. The tables in the report show that while the average number of deaths among 1,000 miners is 18, among 1,000 liquor dealers it is 25, and that the average life of liquor sellers is more than ten years less than that of other men.

* "—a Castilla y a Leon
nuevo mundo dio Colon—"

